# Identification of Species-Specific Peptide Markers in Highly Processed Meat Products Using De Novo Sequencing

**DOI:** 10.3390/foods15132294

**Published:** 2026-06-26

**Authors:** Renata Biba, Mihaela Pravica, Ivana Varenina, Nina Bilandžić, Mario Cindrić

**Affiliations:** 1 Laboratory for Bioanalytics, Division of Molecular Medicine, Ruđer Bošković Institute, Bijenička Cesta 54, 10000 Zagreb, Croatia; renata.biba@irb.hr (R.B.); mihaela.pravica@irb.hr (M.P.); 2 Laboratory for Residue Control, Department of Veterinary Public Health, Croatian Veterinary Institute, Savska Cesta 143, 10000 Zagreb, Croatia; kurtes@veinst.hr (I.V.); bilandzic@veinst.hr (N.B.)

**Keywords:** meat adulteration, species identification, de novo peptide sequencing, peptide derivatization, data independent acquisition, mass spectrometry

## Abstract

Processed meat products represent a major challenge for proteomic species identification due to extensive thermal treatment and protein structural changes. In this study, species-specific peptides in pork, chicken, and bovine meat products were identified using a directed fragmentation-assisted de novo sequencing workflow that combines 4-formylbenzene-1,3-disulfonic acid (FBDA) peptide derivatization, dual-polarity data-independent mass spectrometry (DIA-MS), and Protein Acrobat de novo sequencing software. Comparative analysis of non-fractionated and strong cation exchange (SCX)-fractionated pork luncheon samples improved peptide and protein identification after fractionation, with 312 peptides and 115 protein groups detected exclusively in fractionated samples. Species-specific peptides were predominantly assigned to conserved muscle-related proteins, including myosin, troponin, and tropomyosin, while sequence variability enabled reliable species discrimination despite protein conservation across species. To evaluate applicability for food fraud detection, mixed meat samples containing 10% chicken in pork or bovine matrices were analyzed, reflecting potential economically motivated adulteration through substitution with lower-cost meat components. Several chicken-specific peptides remained detectable in both mixtures, demonstrating robustness of the FBDA-assisted peptide sequencing combined with SCX fractionation and DIA-MS for detection of adulteration in complex processed food matrices. These findings establish a mass spectrometry-driven orthogonal method to ELISA testing for fast, reliable and accurate metaproteome analysis of highly processed food.

## 1. Introduction

Croatian food safety regulations, fully aligned with EU standards, require accurate labeling and species traceability to protect consumers, yet a recent study on seafood labeling has revealed persistent misidentification challenges [1]. These fraudulent practices undermine consumer trust and regulatory enforcement, a pattern also observed in meat adulteration, particularly within highly processed meat products that present significant economic and public health risks to large populations [2]. Such adulteration often involves blending cheaper undeclared mammals, plants, or even fish into products marketed as “pure” beef, pork, or defined blends, leaving the true composition, extent of adulteration, and health hazard levels unclear. Addressing this issue necessitates the development of highly precise, specific, and sensitive advanced methods for accurate detection and detailed compositional analysis of food products [3,4].

A range of technologies is used to combat food adulteration, including physicochemical analyses, biochemical methods, spectroscopy in general and especially nuclear magnetic resonance (NMR) spectroscopy [5], isotope mass spectrometry (MS) [6], and, most commonly, DNA-based quantitative polymerase chain reaction (qPCR) assays [4,7]. While qPCR is highly selective and reliable for single-species confirmation, it is limited by restricted multiplexing capacity, higher analytical costs for multi-target detection, and a lack of robust quantitative capability for determining the extent of adulteration in a food product. Advanced methodologies previously established, such as the MEATiCode proteomic liquid chromatography-tandem mass spectrometry (LC-MS/MS) approach [8], and high-resolution MS combined with a proteogenomics strategy [9] enable simultaneous identification of multiple species by targeting stable peptide markers in highly processed foods, thus overcoming key limitations of conventional DNA-based assays, such as DNA degradation under harsh processing conditions. The mentioned method allows high-throughput, multiplex authentication even in complex meat mixtures.

Proteomics is increasingly recognized as a key methodology for objectively determining the composition and authenticity of food, particularly in the case of processed meat products [10,11,12,13,14,15]. Based on bottom-up proteomic approaches and LC-MS/MS analyses, sets of species-specific, heat-stable peptide markers have been identified, enabling reliable identification of meat species in complex, thermally processed food products [3]. In addition to these qualitative methods [3], quantitative methods, such as those employing stable isotope labeling, have been utilized for meat authentication; however, these approaches are often limited by their capacity for multi-protein composition identification and high operational costs [16,17]. Conversely, label-free MS quantification and the integration of non-targeted proteomics have broadened the analytical scope of meat authenticity studies. These label-free strategies not only support the detection of adulteration but also enable a nuanced evaluation of qualitative differences, compositional variations, and the precise extent of species substitution across diverse meat products [12,18].

In this context, our recently developed bottom-up proteomics approach based on matrix-assisted laser desorption/ionization—time of flight/time of flight (MALDI-TOF/TOF) tandem MS coupled with de novo peptide sequencing has demonstrated that bacterial species can be rapidly and accurately identified in complex biological samples, bypassing the need for small, predefined peptide-mass libraries [19]. Although MALDI-MS has demonstrated suitability for rapid identification workflows, LC-ESI-based proteomics is more widely applied both in data-dependent (DDA) and data-independent acquisition (DIA) due to improved sensitivity, reduced matrix effects, and chromatographic separation that enhances peptide coverage in complex samples. Accordingly, we aimed to extend the developed approach towards nano-ESI-based DIA-MS [9,20]. Recent advances in de novo sequencing algorithms, including deep-learning-based transformer models for de novo sequencing of DIA-MS data [21] and fully probabilistic de novo sequencing approaches [22], enhanced the accuracy of peptide-sequence assignment without relying solely on predefined spectral libraries. By shifting the focus from limited spectral libraries to broad-scale database searches of heat-stable peptide markers, this approach provides a more flexible and comprehensive framework for resilient, routine analytical protocols to verify the authenticity of highly processed products. In this context, de novo sequencing can serve as a complementary strategy, particularly by supporting peptide identification in cases where database annotation is incomplete or ambiguous, and by providing sequence information without prior specification of the target species [23]. This is especially relevant for highly processed foods, where extensive protein degradation and matrix complexity can obscure peptide signals and complicate reliable inference of all contributing species, including potential undeclared or contaminating components. Therefore, it enables the identification of processing-stable peptides that persist through food processing and may serve as potential species-specific biomarkers for authentication of highly processed food products.

**Figure 1:**
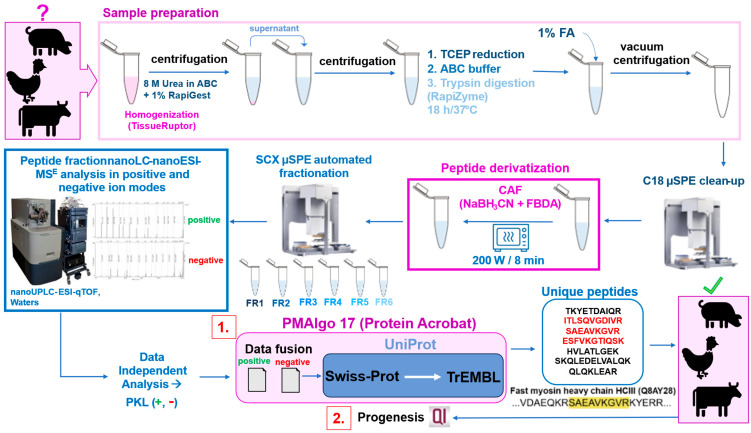
Workflow for species identification in complex meat mixtures by de novo peptide sequencing and data-independent acquisition mass spectrometry (DIA-MS). Meat luncheon samples (pig, bovine, chicken) were processed, FBDA-derivatized, and SCX-fractionated. Fractions were analyzed by nanoUPLC–ESI–QTOF (positive/negative modes). MS^E^ data were processed in PLGS and combined in PMAlgo 17 for graph-based de novo sequencing, followed by UniProt-based species marker identification and Progenesis QI software (v3.0, Waters Corporation, Milford, MA, USA) quantification of validated markers in mixed samples. SPE = solid phase extraction; ABC = ammonium bicarbonate; CAF = chemically activated fragmentation; FA = formic acid; TCEP = tris(2-carboxyethyl)phosphine; FBDA = 4-formylbenzene-1,3-disulfonic acid; SCX = strong cation exchange; PLGS = ProteinLynx Global Server; nanoLC = nano liquid chromatography; nanoESI = nano electrospray ionization; MS^E^ = data-independent MS/MS acquisition; nanoUPLC = nano ultra-performance liquid chromatography; qTOF = quadrupole time-of-flight mass spectrometer; PMAlgo 17 = a component of the Protein Acrobat software.

Our laboratory utilizes a specialized *N*-terminal peptide derivatization protocol with 4-formylbenzene-1,3-disulfonic acid (FBDA) [24], optimized for nanoESI-DIA, which serves as a critical step in ensuring the comprehensive detection of species-specific marker peptides. In this context, peptide derivatization represents a critical optimization step that enhances detectability and sequence assignability, enabling robust recovery of marker peptides and minimizing ion loss during analysis [24]. Moreover, it enables MS analysis in both positive- and negative-ion mode, increasing the peptide coverage and improving de novo sequencing robustness in complex, processed meat products where ionization behavior differs between modes. In this context, the aim of the present study was to employ this integrated FBDA-assisted nanoLC-ESI-MS workflow, combined with custom-developed de novo sequencing software named Protein Acrobat, to identify species-specific peptide markers in highly processed chicken, pork, and bovine samples, and to subsequently verify the presence of those markers in complex mixed meat samples (Figure 1).

## 2. Materials and Methods

### 2.1. Chemicals and Materials

Ammonium bicarbonate (ABC), formic acid (FA), leucine enkephalin (LE), urea, and isopropanol were obtained from Sigma-Aldrich (St. Louis, MO, USA). Acetonitrile (ACN), ammonium acetate, 4-formylbenzene-1,3-disulfonic acid (FBDA) disodium salt hydrate, tris(2-carboxyethyl)phosphine (TCEP), and sodium chloride (NaCl) were obtained from Merck Millipore (Darmstadt, Germany). Sodium cyanoborohydride (NaBH_3_CN) was purchased from G Biosciences (St. Louis, MO, USA). Ammonium formate was obtained from Honeywell (Charlotte, NC, USA). RapiGest SF and RapiZyme Trypsin were purchased from Waters (Milford, MA, USA). Ultrapure water (18 MΩ·cm) was produced in-house using Milli-Q system Direct-Q^®^ 3UV from Merck Millipore. Resin-free AssayMAP cartridges (5 μL; Agilent Technologies, St. Clara, CA, USA) were packed in-house with non-standardized sorbents using a dry-fill high-pressure method. Briefly, the weighed material was loaded onto each cartridge and compressed with air at 2.5 bar. Sepra^TM^ C18-E (50 µm, 65 Å) was obtained from Phenomenex (Torrance, CA, USA) and Bondesil SCX (40 µm, 300 Å) was obtained from Agilent Technologies (St. Clara, CA, USA).

### 2.2. ELISA Analysis of Meat Species Proteins

Species-specific proteins in cooked and processed meat samples were determined using the ELISA-TEK^®^ Cooked Meat Species Assay (Cat. No SE110009, ELISA Technologies, Gainesville, FL, USA) according to the manufacturer’s instructions. The kit was used for the detection of beef, pork, and poultry proteins in cooked and preserved meat products at detection levels of 1–4%. The assay is based on a competitive ELISA format using antibodies specific to species-characteristic glycoproteins.

Unprocessed meat samples were heated first at 95–100 °C for 15 min using an ultrasonic bath (Grant Instruments, Cambridge, UK). Meat samples (5 g) were ground and homogenized in 10 mL of 0.9% (*w*/*v*) NaCl solution using a Waring Commercial Blender 7011HS (Waring Commercial, Stamford, CT, USA) for 1 min. Samples were incubated for 1 h at room temperature and centrifuged at 3000× *g* for 10 min at room temperature using a Rotanta 460R centrifuge (Hettich Zentrifugen, Tuttlingen, Germany); the aqueous layer was filtered through Whatman^®^ Grade 4 filter paper (Merck KgaA, Darmstadt, Germany) and pH-adjusted to 6.0–8.0.

For the assay, 100 μL of sample extract, negative controls (positive controls for other species), positive controls, and 1% positive controls were added to streptavidin-coated wells and incubated at room temperature for 60 min with vigorous shaking, followed by three washes with washing solution. 25 μL of species-specific biotinylated antibody was added and incubated for 60 min at room temperature, followed by three washes; then, 25 μL of streptavidin–peroxidase conjugate was added and incubated for 30 min with six subsequent washes. 50 μL of ABTS substrate was added and incubated for 30 min at room temperature, reactions were stopped by adding 50 μL stop solution, and absorbance was measured at 414 nm (subtracting reference wavelength 492 nm) using a Sunrise-Basic Tecan absorbance microplate reader (Tecan Austria GmbH, Grödig, Austria). Optical density (OD) values were determined by subtracting the mean blank OD from the mean sample OD values for the respective species. The test was considered valid when the positive control OD exceeded eight times the negative control OD. The presence of the tested species was confirmed when sample OD ≥ 1% positive control OD, and the sample OD was greater than the sum of the negative control OD and three times the standard deviation (3 × SD) of the negative controls, as represented in Table 1.

### 2.3. Sample Preparation for Mass Spectrometry

For each analysis, single-species meat luncheon loaf samples (chicken, beef, and pork), as well as mixed samples containing 10% chicken and 90% beef or pork luncheon loaf, were processed. Briefly, 100 mg of each sample was homogenized in 550 μL of extraction buffer containing 8 M urea in 50 mM ABC (pH 7.8) supplemented with 0.1% (*w*/*v*) RapiGest SF (Figure 1). Homogenization was performed using a TissueRuptor (Qiagen, Hilden, Germany) in five cycles of 30 s, with 30 s cooling intervals on ice between cycles. The resulting homogenate was incubated at 37 °C for 30 min to enhance protein solubilization and extraction efficiency and centrifuged at 15,000× *g* for 20 min at 10 °C. The supernatant was transferred to a clean tube and proteins were reduced with 10 mM TCEP for 30 min. Prior to digestion, the sample was diluted 5-fold with 50 mM ABC buffer (pH 7.8). Proteolytic digestion was carried out using RapiZyme Trypsin at an enzyme-to-protein ratio of 1:20 (*w*/*w*) for 18 h at 37 °C. Reaction was quenched by adding FA to a final concentration of 1% (*v*/*v*), which simultaneously precipitated residual RapiGest surfactant. Precipitated material was removed by centrifugation.

### 2.4. Peptide Clean-Up

The obtained peptide mixture was desalted and concentrated using the AssayMAP Bravo Platform (Agilent Technologies, St. Clara, CA, USA) for positive-pressure micro-solid-phase extraction (PP-µSPE) equipped with in-house packed C18-E cartridges. Prior to sample loading, cartridges were conditioned with 100 μL of 50% ACN containing 0.1% FA at a flow rate of 300 μL min^−1^. Subsequently, 100 μL of peptide digest was loaded onto the cartridges at 10 μL min^−1^. Bound peptides were washed with 0.1% FA and eluted with 25 μL of 80% ACN containing 0.1% FA at a flow rate of 10 μL min^−1^.

### 2.5. Peptide Derivatization

Peptide derivatization procedure was done as previously described [24]. Vacuum-dried peptide samples were reconstituted in 30 μL of freshly prepared derivatization solution containing 23.5 mM FBDA and 95.5 mM NaBH_3_CN in 10 mM potassium dihydrogen phosphate buffer (pH 5.0). Derivatization was carried out in a household microwave oven at 200 W for 8 min. Following derivatization, samples were diluted in equilibration buffer compatible with SCX fractionation consisting of 1% FA in 25% ACN.

### 2.6. Peptide Fractionation

Peptide fractionation was performed on the AssayMAP Bravo Platform (Agilent Technologies, St. Clara, CA, USA) using in-house packed SCX PP-µSPE cartridges, as previously described [25]. Prior to sample loading, cartridges were conditioned with 100 μL of 400 mM ammonium formate containing 1% FA in 25% ACN at a flow rate of 300 μL min^−1^. A total of 100 μL of derivatized peptide solution was loaded onto the cartridges and washed with 1% FA in 25% ACN to remove salts and other contaminants. Fractionation was subsequently carried out using a stepwise pH-gradient elution with six buffers of increasing pH, each prepared in 25% ACN: 40 mM ammonium formate (pH 3.5), 40 mM ammonium formate (pH 4.0), 40 mM ammonium acetate (pH 4.5), 40 mM ammonium acetate (pH 5.0), 40 mM ammonium acetate (pH 6.0), and 100 mM ammonium hydroxide (pH 9.5). Fractions obtained from each elution step were collected separately, vacuum dried, and stored at −80 °C prior to LC–MS analysis.

### 2.7. Nano-Ultra-Performance Liquid Chromatography Coupled with Electrospray Ionization Quadrupole Time-of-Flight Mass Spectrometry (nanoUPLC-ESI-QTOF-MS) Analysis

Dried peptide fractions were reconstituted in 20 μL of 0.1% FA for the LC-MS^E^ analysis. Peptides were separated on an ACQUITY UPLC M-Class chromatography system equipped with an Acquity UPLC 2G-V/M symmetry C18 trap column (100 Å, 5 μm, 180 μm  ×  20 mm) and an Acquity UPLC BEH C18 analytical column (130 Å, 1.7 μm, 100 μm  × 100 mm) (Waters, Milford, MA, USA). The column temperature was set to 40 °C. Injection volume was set to 2.5 μL for all samples. Mobile phase A consisted of aqueous 0.1% FA (*v*/*v*) in Milli-Q water, and mobile phase B consisted of 0.1% (*v*/*v*) FA in 95% (*v*/*v*) ACN. Isocratic delivery of solvent A into the trap column was performed at a flow rate of 15 μL min^−1^ for 2 min. The samples were eluted under gradient elution conditions at a flow rate of 1 μL min^−1^ and a run time of 32 min. The following elution gradient was used: 0–3 min, 80% solvent A; 3–24 min, 45% solvent A; 24–27 min, 1% solvent A; 27–29 min, 80% solvent A; and 29–32 min, 80% solvent A. The modifier solution of 1 mM ethyl methanoate in isopropanol was introduced from a Synapt A channel by a “T” connector at a flow rate of 0.4 μL min^−1^.

A nanoUPLC system was coupled to a nanoESI-QTOF Synapt G2-Si mass spectrometer (Waters, Milford, MA, USA), and instrument parameters were set using MassLynx software (v4.1, Waters). The MS and MS^E^ data were acquired in resolution mode. Mass range was set to 500–2500 *m*/*z* for MS survey, 50–2500 *m*/*z* for MS^E^ acquisition in positive ion mode and 50–4200 *m*/*z* for negative ion mode. The ion source parameters were set as follows: nitrogen flow was 1.1 bar with a source temperature of 80 °C, and the capillary voltage was set to 4.2 kV in the positive ion mode and 3.2 kV in the negative ion mode. In positive ion mode, the cone voltage was set to 40 V, while collision energy was maintained at 4 V for the low-energy function and ramped from 20 to 45 V for the high-energy function. In negative ion mode, the cone voltage was increased to 80 V, whereas collision energy settings remained at 4 V for the low-energy function and 20–45 V for the high-energy function. Spectra were acquired with a scan time of 1 s for both MS and MS^E^ analyses. Mass accuracy was maintained by continuous infusion of 1 ng/μL of leucine enkephalin prepared in 50% (*v*/*v*) isopropanol containing 0.1% (*v*/*v*) FA as the lock-mass calibrant.

### 2.8. Data Analysis

MS^E^ data acquired in both positive and negative ionization modes were processed using ProteinLynx Global Server (PLGS, version 3.0.3, Waters, Milford, MA, USA) and exported as peak list (PKL) files. Data obtained from individual SCX fractions both ionization modes were combined into a single dataset using PMAlgo 17 (Protein Acrobat, Consius, Zagreb, Croatia; [19]), which calculates the most probable peptide sequences from fragmentation patterns by aligning negative MS/MS and positive MS/MS mass spectra using a graph-based algorithm. A total of 6 SCX fractions were analyzed per sample.

For determination of the most probable peptide sequences using de novo sequencing, the following parameters were applied: a top pairs percentage at 10% (selecting the 10% most intense spectra separately in positive and negative modes), a top ions percentage at 10% (representing the 10% most intense spectra after combining both ionization modes), mass tolerance of 0.01 Da, precursor percentage after processing of 20%, maximum of 20 sequences per spectrum, maximum Δpm of 0.01 Da (excluding any matches with mass errors greater than 0.01 Da), minimum sequence length of 8 amino acids, minimum sequence length of 9 amino acids for taxonomic assignment, and maximum σ count of 20 for statistical mass processing.

After applying these filtering criteria, the resulting peptide sequences were searched against the UniProtKB/Swiss-Prot for the identification of unique, species-specific peptide markers. In the case of mixed meat samples, unique peptides were first validated in pure meat samples through searching against the UniProtKB/Swiss-Prot database. Following validation, the corresponding peptides were further analyzed using Progenesis QI for proteomics (v3.0, Waters Corporation, Milford, MA, USA) in positive ion mode to enable confirmation of species origin within the complex mixtures. Experimental spectra were matched against theoretical spectra derived from the reference proteomes of *Bos taurus* (UniProt ID: UP000009136), *Sus scrofa* (UniProt ID: UP000008227), and *Gallus gallus* (UniProt ID: UP000000539). For Progenesis QI database searches, peptide and fragment ion mass tolerances were set to automatic, and the false discovery rate (FDR) threshold was maintained below 1%. FBDA (mass increment 249.96059 Da) was set as a fixed modification on peptide *N*-termini, and methionine oxidation was considered as a variable modification during database search. Protein identification criteria required a minimum of three fragment ions per peptide, seven fragment ions per protein, and at least one unique peptide per protein. Due to the limitations of Progenesis QI software regarding negative-ion mode MS data processing, complementary analyses were performed in PLGS using the same identification criteria. Finally, unique peptides were assigned to specific proteins using the UniProt/TrEMBL database through PMAlgo 17 software, with only proteins exhibiting the highest coverage scores included in Table 2.

## 3. Results

### 3.1. ELISA Screening of Single-Species Meat Products

Initial screening for beef, pork, and poultry proteins in commercially available single-species processed meat products was performed using the ELISA-TEK Cooked Meat Species kit (ELISA Technologies, Gainesville, FL, USA), a double sandwich ELISA method with a reported detection limit of 1–4% protein content and validated for 2% contamination level. The validity of the assay was confirmed using negative controls, 1% and 100% positive controls, and laboratory-prepared processed meat controls. Beef- and pork-specific controls met the validation criteria across all samples (Table 1), and corresponding beef and pork proteins were confirmed in analysed beef and pork luncheon samples in accordance with product declarations. In contrast, chicken proteins were inconsistently detected in chicken luncheon samples, as the measured OD values failed to exceed the 1% positive-control threshold, except in chicken parboiled sausage. Notably, this chicken parboiled sausage also yielded a positive signal for pork proteins, suggesting limited assay specificity for this product. Additionally, one beef sample (beef luncheon) tested positive for pork proteins alongside beef proteins. Overall, these results indicate that the ELISA assay performed reliably for beef- and pork-containing products under the conditions tested, but its performance was less consistent for poultry in highly processed matrices. This underscores the need for alternative and orthogonal methods that provide more specific and reliable detection of species-specific proteins in complex, highly processed meat samples.

### 3.2. Effect of FBDA Derivatization on Peptide Identifications in Data-Independent Acquisition Mass Spectrometry Analysis in Positive and Negative Ion Mode

**Figure 2:**
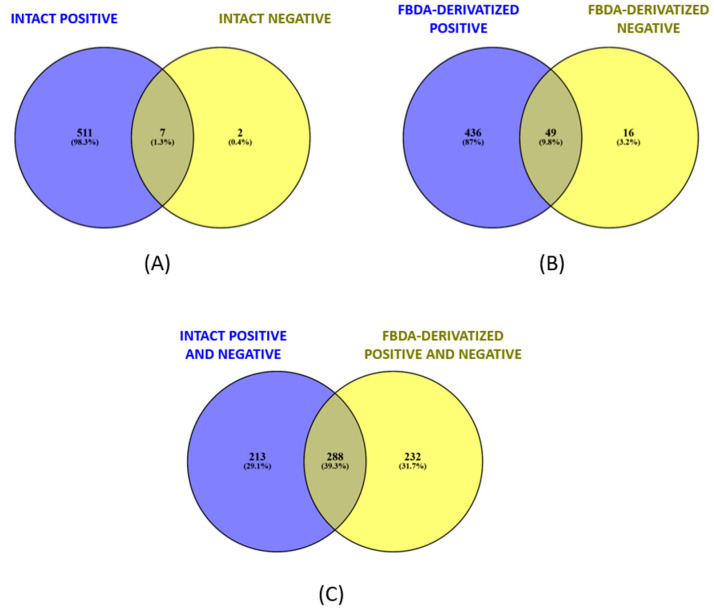
Venn diagrams showing the number of identified peptides in intact (**A**) and FBDA-derivatized samples (**B**) in positive and negative ion modes, as well as combined positive and negative ion mode data for intact and FBDA-derivatized peptides (**C**) following DIA-MS^E^ analysis. Data are derived from pork luncheon samples. Overlapping regions indicate shared peptide identifications between sample preparation strategies, while non-overlapping regions represent condition-specific identifications.

Given the limitations of ELISA for detecting species-specific proteins in highly processed meat products, as highlighted in the previous section, liquid chromatography (LC) coupled with data-independent acquisition mass spectrometry (DIA-MS) was employed as the main analytical platform for proteomic-based species identification in processed meat samples (chicken, pork and beef luncheon loaf). To evaluate the performance of the FBDA.assisted peptide identification workflow, in initial comparison was performed between intact and FBDA-derivatized peptides (Figure 2).

In negative ion mode, FBDA derivatization resulted in an increased number of identified peptides compared to the non-derivatized counterpart (65 and 9 identified peptides, respectively). Although the total number of peptide identifications did not differ significantly between derivatized and non-derivatized samples when considering both ionization modes (501 in the intact sample and 520 in the FBDA-derivatized sample), Venn diagram analysis revealed that only ~40% of peptides were shared between the two approaches. This indicates that FBDA derivatization not only enhances peptide detectability but also expands the diversity of the peptide population accessed by DIA-MS analysis.

### 3.3. Effect of SCX Fractionation on Peptide and Protein Identifications in Data-Independent Acquisition Mass Spectrometry Analysis in Positive and Negative Ion Mode

**Figure 3:**
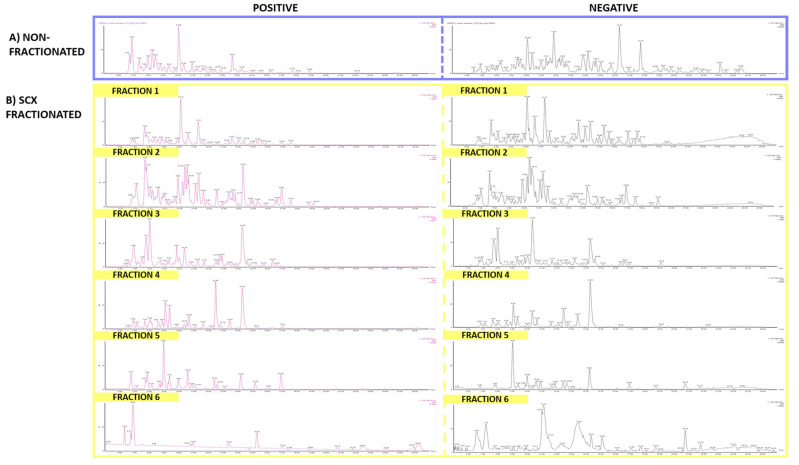
Base peak chromatograms (BPC) of FBDA-derivatized peptides from pork luncheon loaf, analyzed by DIA-MS^E^ in both positive and negative ion modes. (**A**) Non-fractionated sample, (**B**) SCX-fractionated samples (fractions 1–6).

To assess the impact of sample fractionation on analytical performance of the analysis, peptide and protein identifications were compared between non-fractionated and strong cation exchange (SCX) fractionated FBDA-derivatized peptides obtained from pork luncheon samples (Figure 3). SCX fractionation reduced sample complexity by distributing peptide signals across fractions, in both positive and negative ion modes, facilitating detection of a broader range of peptide species and enabling more comprehensive peptide and protein identification.

**Figure 4:**
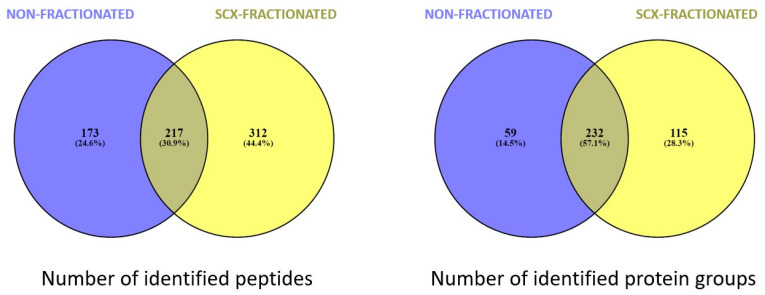
Venn diagrams showing the number of identified peptides (**left**) and protein groups (**right**) in non-fractionated and SCX-fractionated samples (from 6 SCX fractions) following DIA-MS^E^ analysis in positive and negative ion modes combined. The data represent FBDA-derivatized peptides obtained from pork luncheon samples. Overlaps indicate shared identifications between sample preparation strategies, while unique fractions represent condition-specific identifications.

Specifically, at the peptide level, SCX fractionation yielded 312 peptides specific for that approach, compared to 173 peptides specific to the non-fractionated workflow, with 217 peptides common to both methods. Similarly, at the protein group level, 115 protein groups were specific to the fractionated sample, 59 were specific to the non-fractionated sample, and 232 were identified in both (Figure 4). Overall, SCX fractionation increased the number of specific FBDA-derivatized peptide and protein identifications while maintaining substantial overlap with the non-fractionated dataset, confirming its utility in identification of species-specific peptides.

Pork luncheon samples were used as a representative dataset for initial evaluation of SCX fractionation performance compared to non-fractionated workflow. Overlaps represent identifications common to both sample preparation strategies (non-fractionated and fractionated), while unique portions correspond to condition-specific identifications, illustrating the enhanced proteome depth achieved with SCX fractionation. Based on these findings, SCX fractionation was applied to all remaining single-species and mixed meat samples. Representative chromatograms of fractionated samples in both positive and negative ion modes are available in the Supplementary Information (Figures S1–S8).

### 3.4. Identification of Species-Specific Peptides in Single-Species Meat Products with De Novo Peptide Sequencing

A previously developed Protein Acrobat de novo sequencing workflow [19] was applied to FBDA-derivatized peptides from processed meat samples to enable species-specific peptide identification. The workflow follows a sequential approach: (1) merging of positive and negative ion modes MS/MS spectra to increase sequence reliability, (2) Kendrick mass defect (KMD)-based filtering to improve *b*- and *y*-ion assignment accuracy, (3) graph-based algorithm for de novo peptide sequence reconstruction, and (4) database searching against UniProtKB/Swiss-Prot and UniProtKB/TrEMBL to identify species-specific unique peptides. This integrated approach enhances confidence in peptide identifications and facilitates reliable species authentication in complex processed meat matrices. A peptide is classified as species-specific when it meets two criteria: (1) the peptide sequence matches a protein from the target species (e.g., *Sus scrofa* for pork, searched against the UniProtKB/Swiss-Prot database), and (2) the identical peptide sequence is absent from all other species in the UniProtKB/Swiss-Prot database, including closely related species. UniProtKB/Swiss-Prot is used first for species-specificity determination because it contains manually curated, high-quality, non-redundant sequences with expert-verified annotations. Following species-specific peptide confirmation, corresponding proteins are identified by searching the larger UniProtKB/TrEMBL database, which provides broader sequence coverage, including newly sequenced proteins and isoforms not yet curated into Swiss-Prot. This sequential strategy balances accuracy (Swiss-Prot) with comprehensiveness (TrEMBL), ensuring reliable species authentication while maximizing protein detection in complex processed meat matrices.

Species-specific resilient peptides and their corresponding proteins identified in processed pork, chicken, and bovine meat samples are summarized in Table 2. In total, 6, 13, and 4 species-specific peptides were identified in pork (*Sus scrofa*), chicken (*Gallus gallus*), and bovine (*Bos taurus*) samples, respectively representing 1.1%, 2.5%, and 0.8% of all identified peptides. The processed and thermally treated nature of the meat samples substantially reduced proteome complexity, resulting in selective detection of more stable and highly abundant muscle-related protein families, including myosin, troponin, and tropomyosin. Additionally, pork samples included resilient peptides derived from hemoglobin and creatine kinase, while bovine samples included collagen type I, reflecting contributions from connective tissue proteins. Chicken samples showed the broadest protein diversity, with peptides assigned mainly to myofibrillar proteins and calcium-binding proteins such as parvalbumin. Despite originating from highly conserved protein families across species, sufficient sequence variability enabled clear species-level discrimination. Notably, only half of pork-specific peptides identified in the fractionated workflow were also detected in the non-fractionated dataset (MFLGFPTTK, TYFPHFNLSHGSDQVK, and ALTLEIYKK), confirming limited performance of the non-fractionated workflow (Supplementary Information, Table S1). These species-specific resilient peptides serve as robust diagnostic markers for meat authentication even in heavily processed products in the following experiments, overcoming the limitations of protein-based methods like ELISA.

### 3.5. Detection of Species-Specific Peptides in Mixed Meat Samples

To evaluate the applicability of species-specific resilient peptide markers for meat adulteration detection, luncheon loaf meat mixtures containing 10% chicken combined with either pork or bovine loaf were analyzed by DIA-MS^E^, and the results are summarized in Table 3. In the 10% chicken/90% pork mixture, two chicken-specific peptides (LQDLVDKLQMK and SAEAVKGVR) were detected, while the remaining chicken peptides were not observed. In contrast, a higher number of chicken-derived peptides were detected in the 10% chicken/90% bovine mixture, including SSVFVVHPK, LQDLVDKLQMK, SAEAVKGVR, and HLEEEIKAK. Relative peptide signal intensities were calculated using single-species chicken sample as a reference (100%). In the 10% chicken/90% pork mixture, LQDLVDKLQMK and SAEAVKGVR retained 4.54% and 1.49% of their original signal, respectively. In the 10% chicken/90% bovine mixture, higher residual signals were observed for SSVFVVHPK, LQDLVDKLQMK, SAEAVKGVR, and HLEEEIKAK, corresponding to 7.49%, 11.4%, 1.59%, and 2.57%, respectively.

**Figure 5:**
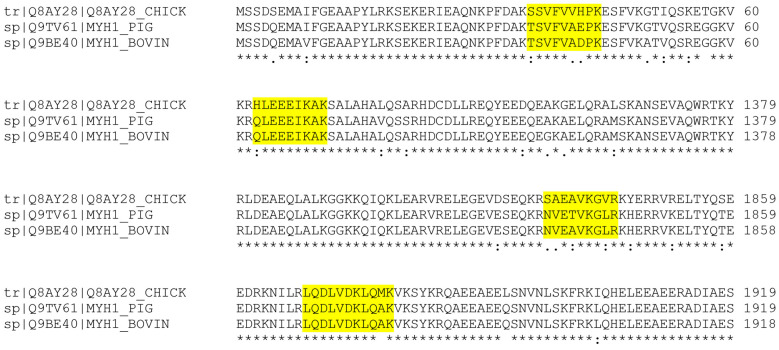
Clustal Omega multiple sequence alignment of selected chicken-specific resilient peptide sequences derived from fast myosin heavy chain HCIII fragments (*Gallus gallus*, UniProt ID: Q8AY28) and homologous regions of myosin I found in pig (*Sus scrofa*; UniProt ID: Q9TV61) and bovine (*Bos taurus*, UniProt ID: Q9BE40). The aligned specific peptide sequences are highlighted. An asterisk (*) denotes positions with a single, fully conserved residue; a colon (:) indicates that some sequences have different amino acids at that position, which have strongly similar chemical properties, and period (.) marks amino acids differ but have moderately similar chemical properties; a blank space ( ) indicates that the amino acids are very different at that position.

Overall, peptides LQDLVDKLQMK and SAEAVKGVR were consistently detected in both mixtures, and multiple sequence alignment of corresponding peptides in pig and bovine proteins confirmed clear sequence differences relative to the chicken homologues (Figure 5), further supporting their specificity and strong potential as robust markers for chicken authentication in complex meat matrices.

## 4. Discussion

Food adulteration, particularly economically motivated food fraud, represents a great challenge for food authenticity and safety, requiring reliable analytical strategies for accurate species identification in complex food matrices [4,9]. The present study demonstrates that mass spectrometry-based proteomics, combined with micro-solid phase extraction (µSPE) strong cation exchange (SCX) fractionation and de novo peptide sequencing, provides a robust and specific method for species authentication in highly processed meat products, overcoming key limitations of immunoassay-based methods, such as ELISA.

Initial ELISA screening using the ELISA-TEK Cooked Meat Species kit confirmed expected beef and pork proteins in most single-species luncheon samples (Table 1), as previously confirmed [26], but showed inconsistent detection of chicken proteins and unexpected cross-reactivity between chicken and pork signals. Chicken proteins were found only in the parboiled sausage sample (one out of four), which also tested positive for pork proteins. One beef sample (beef luncheon) additionally tested positive for pork proteins. These findings indicate that antibody-based detection can be compromised by protein denaturation, fragmentation, and matrix effects in highly processed meats, leading to reduced sensitivity and specificity. This is consistent with previous reports that ELISA, while sensitive and suitable for routine screening of some meat products, may fail to reliably detect species-specific proteins in particular thermally processed and complex food matrices [27,28]. The observed cross-reactivity further underscores the risk of false-positive results when epitopes are altered or when antibodies only recognize conserved regions across species.

Given the constraints of ELISA for processed meat authentication, we turned to de novo peptide sequencing combined with data-independent acquisition mass spectrometry (DIA-MS) as the core proteomic approach for species identification. A key component of the workflow was the application of the *N*-terminal peptide derivatization reagent, 4-formyl-1,3-benzenedisulfonic acid (FBDA), which facilitates chemically activated fragmentation (CAF) in both positive and negative ion modes. This process enhances peptide fragmentation efficiency during collision-induced dissociation (CID) and promotes the generation of informative *b*-ion series, thereby improving sequence elucidation and de novo sequencing performance [19,24,29,30]. FBDA derivatization also enabled peptide analysis in both positive- and negative-ion modes, generating complementary fragmentation patterns and increasing the number of interpretable fragment ions. This resulted in an overall higher number of peptide identifications compared to non-derivatized conditions, particularly in negative ion mode (Figure 2). When positive and negative ion mode data were combined, only ~40% overlap was observed between derivatized and non-derivatized datasets, indicating substantial differences in the peptide populations detected with the two approaches. This reflects the complementary nature of the FBDA-assisted workflow, where derivatization enables access to additional peptide species not efficiently detected without derivatization. The dual-polarity approach also improved sequence confidence and facilitated discrimination between closely related species whose peptide sequences differed by only a few amino acids, as demonstrated in Figure 5. Furthermore, Protein Acrobat, our in-house developed de novo sequencing software, was specifically optimized for FBDA-derivatized peptides through integration of graph-based algorithms and data-filtering procedures designed to exploit characteristic fragmentation behavior of the derivatized peptides. Combined with DIA-MS, which provides improved peptide coverage, reproducibility, and more comprehensive marker detection compared to data-dependent acquisition (DDA)-based workflows, this integrated strategy represents a robust and high-throughput approach for meat authentication in complex processed products.

The sequential database search strategy, using UniProtKB/Swiss-Prot for species-specific peptide determination followed by UniProtKB/TrEMBL for broader protein coverage, balanced accuracy with comprehensiveness. Swiss-Prot is well suited for confirming peptide specificity because its entries are manually curated and non-redundant, reducing the risk of false assignment when evaluating whether a peptide is species-specific. Once uniqueness is established, TrEMBL expands the search space and improves protein annotation by capturing additional sequence records and isoforms that may not yet be curated. This two-step approach balances specificity with coverage and is particularly appropriate for processed food matrices, where confident species discrimination is more important than exhaustive proteome annotation [3].

In addition to the advantages provided by FBDA derivatization and DIA-MS analysis, peptide prefractionation by SCX µSPE further contributed to improved proteome coverage and marker detection. SCX fractionation can increase the likelihood of detecting species-diagnostic peptides through sample complexity reduction [19,31,32], and implementation of automated µSPE protocols ensures reproducibility for high-throughput studies [25,33]. In the present study, fractionation of FBDA-derivatized peptides increased the number of detected peptides and protein groups relative to the non-fractionated workflow (Figure 4), indicating improved sequence recovery and overall proteome coverage. By separating complex peptide mixtures into multiple fractions, the workflow enhanced the detectability of low-abundant and species-specific peptides that would otherwise remain masked in unfractionated sample (Figure 3). This effect was shown in the pork dataset where SCX fractionation revealed 50% more species-specific peptides compared to non-fractionated sample, confirming its utility in species authentication workflows in complex, highly processed meat matrices. Although SCX fractionation increased the overall number of peptide identifications, a subset of peptides was detected exclusively in the non-fractionated sample. This may reflect peptide loss during additional sample handling steps and redistribution of peptides across multiple fractions, which can reduce the abundance of low-intensity peptides within individual fractions and affect their detectability and identification [25]. Importantly, consistency and shown reproducibility of automated µSPE workflows can provide a practical advantage for routine adulteration screening, as future targeted workflows could potentially be simplified by monitoring only selected SCX fractions known to contain species-specific peptide markers, in this way reducing the number of required LC-MS analyses and improving analytical throughput [8,14].

The proposed workflow enabled the detection of candidate species-specific peptide markers for chicken, pig and bovine samples. The distribution of species-specific peptides was consistent with the biochemical properties of processed meat proteins. Most peptide markers originated from abundant and relatively heat-stable muscle proteins, including myosin, troponin, and tropomyosin, which are commonly retained after cooking and therefore remain accessible for MS-based detection [3]. Pork-specific peptides also included sequences from hemoglobin and creatine kinase (Table 2), while bovine samples yielded a collagen-derived marker, suggesting that proteins from sarcoplasmic and connective tissue fractions can also contribute useful authentication peptides when they contain stable diagnostic protein regions [3]. A key strength of the workflow is its ability to distinguish peptides from highly conserved protein families across species (Figure 5). Although many identified proteins belong to homologous muscle protein classes, species-level discrimination was still possible because the detected peptides contained sequence differences sufficient to separate chicken, pork, and bovine homologues. This is a crucial advantage of peptide authentication: instead of depending on intact proteins or antigenic epitopes, the method exploits short sequence variants that remain informative even after heat treatment and processing. Our observation that resilient marker peptides originate mainly from abundant muscle proteins is consistent with reports that myofibrillar and selected sarcoplasmic proteins constitute the primary source of robust peptide markers detectable in thermally treated meat [34], and that these peptides retain diagnostic power even in complex matrices. Analytically, focusing on conserved yet sequence-divergent regions of these major muscle proteins yields high-intensity signals, enhances marker persistence under severe processing conditions, and supports the design of robust targeted LC–MS methods for routine speciation and quantitative adulteration analysis. Within this framework, the present study advances earlier peptide-marker work by demonstrating that directed-fragmentation-assisted de novo sequencing can systematically uncover species-specific peptides from conserved proteins in highly processed luncheon-type matrices, thereby expanding the pool of validated markers available for orthogonal confirmation of meat species identity [34].

Comparison with previously reported heat-stable chicken, pork, and bovine peptide markers (reviewed in [3,35,36]) revealed no direct overlap with the peptide set identified in the present study. These differences likely arise from the analytical approaches used, particularly FBDA derivatization, which alters peptide fragmentation, and DIA-MS^E^ combined with de novo sequencing, which expands peptide detection and yields complementary species-specific markers [3,8,16].

A notable proportion of the identified species-specific peptides contained missed tryptic cleavage sites. This can be attributed to the de novo sequencing workflow, which required a minimum peptide length of nine amino acids for taxonomic assignment. Together with the applied filtering criteria, this favored longer peptide sequences, which in tryptic digests are frequently represented by missed-cleavage products. The prevalence of missed-cleavage peptides may also reflect the highly processed nature of the analyzed meat products. Thermal processing is known to induce structural and chemical changes that affect protein digestibility and protease accessibility, resulting in longer digestion products [37,38]. Importantly, these species-specific peptides were consistently detected across both single-species and mixed-species samples with high confidence. While missed-cleavage peptides may be less suitable as targets for highly standardized quantitative assays, they remain valuable diagnostic markers within a discovery-based workflow, where species specificity and confident taxonomic assignment are the primary criteria for marker selection.

The applicability of the identified peptide markers for adulteration detection was evaluated in mixed meat samples containing 10% chicken combined with either pork or bovine meat (Table 3). Two chicken-specific peptides (LQDLVDKLQMK and SAEAVKGVR) were consistently detected in both mixtures, while additional chicken peptides were detected only in the chicken/bovine mixture. Relative signal intensities of the detected peptides ranged from 1.49% to 11.4% compared to the single-species chicken reference, indicating that even low-intensity peptide signals can be resolved with DIA-MS and de novo sequencing. Collectively, these results demonstrate the method’s sensitivity, indicating that the limit of detection (LOD) may be below the reported 10% level (10% chicken in pork or bovine luncheon meat).

In the broader methodological landscape of LC–MS-based meat authentication, the FBDA-assisted, dual-polarity DIA-de novo sequencing workflow established in this study differs substantially from previously reported approaches in terms of sample preparation, acquisition strategy, and data analysis, and should therefore be considered complementary to existing LC–MS/MS methods rather than a direct replacement. Recent methods such as MEATiCode [8] and related bottom-up LC-MS/MS strategies [39,40] rely on extensive high-resolution discovery experiments to define panels of heat-stable, species-specific tryptic peptides, which are then transferred to targeted MRM or PRM assays to achieve high sensitivity (down to approximately 0.5–1% *w*/*w*) and rapid turnaround in routine testing, mainly in single-polarity positive-ion mode and using relatively simple sample preparation protocols. Similarly, LC-QTOF-MS methods [18] that focus on a limited number of myofibrillar markers or selected signature peptides provide robust detection of specific species in raw and cooked products, but generally assume that candidate markers are already known and that their ionization behavior remains predictable across processing conditions. By contrast, the workflow described here combines SCX prefractionation, *N*-terminal FBDA derivatization, dual-polarity DIA, and graph-based de novo sequencing to expand the accessible marker space in highly processed matrices where peptide modification, aggregation, partial degradation, and non-canonical ionization patterns can reduce the performance of strictly library-driven or targeted methods. This design intentionally trades some of the simplicity and run-time efficiency of one-shot MRM/PRM methods for broader sequence coverage and improved detectability of modified or low-abundance peptides in highly processed matrices, where the stability and ionization behavior of previously defined markers (e.g., myofibrillar peptides selected in earlier LC–MS/MS studies) can be compromised. Accordingly, the main strength of this approach lies upstream of targeted assays, enabling the systematic discovery and verification of resilient species-specific peptides directly in complex and mixed meat products, including markers that are not yet represented in existing databases or LC–MS/MS panels, and thus provides an orthogonal proteomics route to support and refine current LC–MS-based meat authentication workflows.

To further evaluate peptide specificity, UniPept tryptic peptide analysis [41] was performed. The results showed that the suggested peptides are conserved within higher taxonomic levels (e.g., class Aves), indicating that they are not strictly species-exclusive within all avian taxa. Nevertheless, the peptides did not show broad overlap with unrelated food-relevant species and remained informative for distinguishing the target meat species investigated in this study. This suggests that their value lies in practical food authentication contexts rather than absolute species uniqueness.

Overall, this study demonstrates that ELISA screening has limited reliability for species authentication in highly processed meat products, whereas DIA-MS^E^ combined with SCX fractionation and de novo peptide sequencing, without relying on conventional library-based methods, provides a more specific and comprehensive approach. The identification of robust species-specific resilient peptide markers in pork, chicken, and bovine meats, and their successful detection in mixed meat samples, supports the use of proteomic-based workflows for meat authentication and adulteration detection in complex food matrices. These findings contribute to the growing body of evidence that peptide-based mass spectrometry methods are powerful tools for food authentication and regulatory compliance in the meat industry.

## 5. Conclusions

This study shows that FBDA-assisted DIA-MS^E^ combined with SCX fractionation and graph-based de novo peptide sequencing provides a robust and analytically powerful platform for authenticating highly processed meat products. The workflow overcame the limited performance of ELISA in thermally processed samples and enabled the discovery of species-specific peptides from conserved muscle protein families, including myosin, troponin, tropomyosin, and, in some cases, hemoglobin, creatine kinase, collagen, and parvalbumin.

Importantly, the approach proved effective not only in single-species products but also in mixed-meat samples designed to model economically motivated adulteration. The reliable identification of species-specific resilient peptide markers in both pure and adulterated matrices demonstrates the effectiveness of this workflow for food fraud detection, particularly when the adulterant occurs at low concentrations within highly processed samples. In the chicken-containing mixtures, the repeated identification of peptides LQDLVDKLQMK and SAEAVKGVR in both adulterated matrices highlights these peptides as particularly robust diagnostic markers with potential for routine application in species authentication. Adulteration models in this study were restricted to 10% chicken substitution in pork or beef, so the sensitivity of this workflow for detecting lower-level adulteration, more complex mixtures involving three or more undeclared species and non-chicken adulterants remains unresolved. In addition, different thermal treatments and processing (dry-cured, smoked, fermented) or ingredients, such as high-fat, high-salt, or strongly spiced matrices may affect extraction efficiency and ionization, potentially changing the sensitivity of marker detectability, so more complex and diverse fraud scenarios remain to be systematically assessed. The present work was primarily focused on qualitative identification of resilient marker peptides, but rigorous quantitative performance characteristics (limits of detection and reproducibility) have not been fully established and will be necessary before this approach can be adopted as a standardized routine method for meat authentication.

Overall, these findings indicate that combining chemical derivatization, fractionation, and de novo peptide sequencing yields a workflow with greater analytical power than immunoassay-based testing for complex processed meat products. In addition to improving species identification, the approach enhances peptide detectability and confidence in sequence assignment, which is particularly advantageous for challenging food matrices. Future work should expand the marker panel across additional meat species and product types, as well as evaluate performance at lower substitution levels, and further determine the method’s quantitative robustness under varying processing conditions.

## Figures and Tables

**Table 1:** Results of the ELISA screening of processed meat products for the presence of beef, pork, and poultry proteins. A sample was considered positive (POS, +) only if it met all three criteria: A. The optical density (OD) of the sample must be greater than or equal to the OD value of the 1% positive control, as indicated by B. OD > 0.4 assay cutoff (as specified by the manufacturer); C. OD (sample) > OD (max negative) + 3 × SD (negative). Positive-control validity was confirmed when OD (100% positive) ≥ 8 × OD (negative). Each assay used negative controls, 1% and 100% positive controls, and lab-made processed meat controls.

Sample Description	Bovine					Pork					Poultry				
	OD 1% Positive Bovine	OD Sample	A	B	C	OD 1% Positive Pork	OD Sample	A	B	C	OD 1% Positive Poultry	OD Sample	A	B	C
Beef luncheon	1.019	1.254	POS	+	+	0.809	0.073	NEG	−	−	1.016	0.046	NEG	−	−
Beef luncheon	1.339	1.172	POS	+	+	1.172	1.339	POS	+	+	0.771	0.231	NEG	+	+
Beef meat	1.067	1.354	POS	+	+	0.898	0.177	NEG	+	+	1.038	0.059	NEG	−	−
Beef meat	1.067	1.330	POS	+	+	0.898	0.163	NEG	+	+	1.038	0.089	NEG	−	+
Beef minced meat (ćevapi)	1.172	1.430	POS	+	+	0.771	0.121	NEG	+	−	1.026	0.052	NEG	−	−
Beef pie	1.085	1.447	POS	+	+	0.762	0.147	NEG	+	−	0.967	0.103	NEG	+	+
Beef sausage	1.019	1.427	POS	+	+	0.809	0.594	NEG	+	+	1.016	0.060	NEG	−	+
Minced beef meat	1.067	1.370	POS	+	+	0.898	0.157	NEG	+	+	1.038	0.059	NEG	−	−
Chicken parboiled sausage	1.172	0.035	NEG	−	−	0.771	2.935	POS	+	+	1.026	1.632	POS	+	+
Chicken pate	1.067	0.094	NEG	−	−	0.898	0.169	NEG	+	+	1.038	0.649	NEG	+	+
Chicken pate	1.067	0.146	NEG	+	+	0.898	0.134	NEG	+	+	1.038	0.686	NEG	+	+
Chicken pate	1.172	0.051	NEG	−	−	0.771	0.509	NEG	+	+	1.026	0.836	NEG	+	+
Pork ham	1.003	0.032	NEG	−	−	0.734	2.732	POS	+	+	0.928	0.059	NEG	−	−
Pork liver pate	1.172	0.049	NEG	−	−	0.771	2.611	POS	+	+	1.026	0.058	NEG	−	−
Pork liver pate	1.003	0.040	NEG	−	−	0.734	2.733	POS	+	+	0.928	0.052	NEG	−	−
Pork luncheon loaf	1.019	0.027	NEG	−	−	0.809	2.410	POS	+	+	1.016	0.049	NEG	−	−
Pork parboiled sausage	1.067	0.032	NEG	−	−	0.898	2.510	POS	+	+	1.038	0.051	NEG	−	−
Pork—mortadella	1.067	0.030	NEG	−	−	0.898	2.562	POS	+	+	1.038	0.083	NEG	−	+

**Table 2:** Species-specific proteins and unique resilient peptides detected in single-species processed meat products from pig (*Sus scrofa*), chicken (*Gallus gallus*), and bovine (*Bos taurus*). The table lists protein names, UniProtKB/TrEMBL accession numbers, sequence coverage percentages, and unique peptide sequences identified following SCX fractionation (FR1–FR6). Positive detection (+) indicates the peptide was identified in the corresponding fraction, while (−) indicates absence. Scientific names are based on UniProt genome entries (UP000008227 for pig, UP000000539 for chicken, UP000009136 for bovine). The collagen alpha-1(I) chain accession P02453* indicates a reviewed UniProtKB/Swiss-Prot entry; all other entries are from UniProtKB/TrEMBL. Proteins with the highest sequence coverage following UniProtKB/TrEMBL database search are listed.

Species (UniProt ID)	Protein	Accession No. (UniProtKB/TrEMBL)	Sequence Coverage (%)	Resilient Peptide Sequence	FR1	FR2	FR3	FR4	FR5	FR6
Pig—*Sus scrofa* (UP000008227)	Hemoglobin subunit alpha	A0A4X1UIB6	26.24%	MFLGFPTTK	+	−	−	−	−	−
				VGGQAGAHGAEALER	+	−	−	−	−	−
				TYFPHFNLSGSDQVK	−	−	−	−	+	+
	Troponin T, fast skeletal muscle	A0A287AFK2	3.35%	QKYDIINLR	+	−	−	−	−	−
	Myosin-2	A0A8D1L3D5	0.57%	QAYTQQIEELKR	+	−	−	−	−	−
	Creatine kinase	A0A8D1L9L8	2.24%	ALTLEIYKK	+	−	−	−	−	−
Chicken—*Gallus gallus* (UP000000539)	Myosin light chain, phosphorylatable, fast skeletal muscle	A0A8V0WZV4	14.88%	LKGADPEDVIMGAFK	+	−	−	−	−	−
				VLDPDGKGSIKK	−	−	+	−	−	−
	Fast myosin heavy chain HCIII	Q8AY28	2.94%	ESFVKGTIQSK	+	−	−	−	−	−
				SSVFVVHPK	−	+	−	−	−	−
				LQDLVDKLQMK	+	−	−	−	−	−
				SAEAVKGVR	+	+	−	−	−	−
				VRELEGEVDSEQKR	−	−	+	−	−	−
				HLEEEIKAK	−	−	+	−	−	−
	Parvalbumin, muscle	A0A8V0YQ28	7.55%	FFEMVGLKK	−	+	−	−	−	−
	Myosin, light chain 1, alkali; skeletal, fast	A0A8V0Y563	6.41%	ITLSQVGDIVR	+	−	−	−	−	−
	Troponin T3, fast skeletal type	A0A8V1A6S0	3.36%	KKYEIVTLR	−	−	−	+	−	−
	Collagen type I alpha 2 chain	A0A8V0X994	0.79%	GLRGDVGPVGR	+	+	−	−	−	−
	Tropomyosin 2	A0A8V0Y915	5.07%	KATDAEAEVASLNRR	−	−	+	−	−	−
Bovine—*Bos taurus* (UP000009136)	MLY1 protein	Q08E10	7.95%	QQQDEFKEAFLLFDR	+	−	−	+	−	−
	Collagen alpha-1(I) chain	P02453*	1.44%	SGDRGETGPAGPAGPIGPVGAR	+	−	−	−	−	−
	Troponin T fast skeletal muscle type	Q8MKH7	3.20%	QKYDITNLR	+	−	−	−	−	−
	Myosin heavy chain 2	F1MRC2	0.52%	NALAHGLQSAR	−	+	−	−	−	−

**Table 3:** Detection and relative intensity of unique chicken (*Gallus gallus*) resilient peptides in mixed meat samples. The table summarizes the presence (+) or absence (−) of each chicken-specific resilient peptide in two meat mixtures: 10% chicken/90% pig and 10% chicken/90% bovine. Relative intensities were calculated as a percentage (%) of peptide intensity in mixed samples relative to corresponding single-species chicken sample.

Unique Peptide Sequence for Chicken (*Gallus gallus*)	Detection of Unique Chicken Resilient Peptides in Mixture 10% Chicken/90% Pig (Progenesis Software, Positive Ion Mode)	Relative Intensity (%)	Detection of Unique Chicken Resilient Peptides in Mixture 10% Chicken/90% Bovine (Progenesis Software, Positive Ion Mode)	Relative Intensity (%)
LKGADPEDVIMGAFK	−	−	−	−
VLDPDGKGSIKK	−	−	−	−
ESFVKGTIQSK	−	−	−	−
SSVFVVHPK	−	−	+	7.49
LQDLVDKLQMK	+	4.54	+	11.40
SAEAVKGVR	+	1.49	+	1.59
VRELEGEVDSEQKR	−	−	−	−
HLEEEIKAK	−	−	+	2.57
FFEMVGLKK	−	−	−	−

## Data Availability

The mass spectrometry data generated in this study have been deposited in the MassIVE repository under accession number MSV000102216 and are publicly available via doi:10.25345/C5Z892V69.

## Supplementary Materials

The following supporting information can be downloaded at https://www.mdpi.com/article/10.3390/foods15132294/s1: Figure S1. Representative base peak intensity (BPI) chromatograms of FBDA-derivatized peptides obtained from chicken luncheon loaf samples, SCX fractionated, and analyzed by DIA-MS^E^ in positive ion mode. (A) fraction 1, (B) fraction 2, (C) fraction 3, (D) fraction 4, (E) fraction 5, (F) fraction 6; Figure S2. Representative base peak intensity (BPI) chromatograms of FBDA-derivatized peptides obtained from chicken luncheon loaf samples analyzed by DIA-MS^E^ in negative ion mode. (A) fraction 1, (B) fraction 2, (C) fraction 3, (D) fraction 4, (E) fraction 5, (F) fraction 6; Figure S3. Representative base peak intensity (BPI) chromatograms of FBDA-derivatized peptides obtained from beef luncheon loaf samples, SCX fractionated, and analyzed by DIA-MS^E^ in positive ion mode. (A) fraction 1, (B) fraction 2, (C) fraction 3, (D) fraction 4, (E) fraction 5, (F) fraction 6; Figure S4. Representative base peak intensity (BPI) chromatograms of FBDA-derivatized peptides obtained from beef luncheon loaf samples analyzed by DIA-MS^E^ in negative ion mode. (A) fraction 1, (B) fraction 2, (C) fraction 3, (D) fraction 4, (E) fraction 5, (F) fraction 6; Figure S5. Representative base peak intensity (BPI) chromatograms of FBDA-derivatized peptides obtained from 10% chicken/90% pork luncheon loaf samples, SCX fractionated, and analyzed by DIA-MS^E^ in positive ion mode. (A) fraction 1, (B) fraction 2, (C) fraction 3, (D) fraction 4, (E) fraction 5, (F) fraction 6; Figure S6. Representative base peak intensity (BPI) chromatograms of FBDA-derivatized peptides obtained from 10% chicken/90% pork luncheon loaf samples analyzed by DIA-MS^E^ in negative ion mode. (A) fraction 1, (B) fraction 2, (C) fraction 3, (D) fraction 4, (E) fraction 5, (F) fraction 6; Figure S7. Representative base peak intensity (BPI) chromatograms of FBDA-derivatized peptides obtained from 10% chicken/90% beef luncheon loaf samples, SCX fractionated, and analyzed by DIA-MS^E^ in positive ion mode. (A) fraction 1, (B) fraction 2, (C) fraction 3, (D) fraction 4, (E) fraction 5, (F) fraction 6; Figure S8. Representative base peak intensity (BPI) chromatograms of FBDA-derivatized peptides obtained from 10% chicken/90% beef luncheon loaf samples, SCX fractionated, and analyzed by DIA-MS^E^ in negative ion mode. (A) fraction 1, (B) fraction 2, (C) fraction 3, (D) fraction 4, (E) fraction 5, (F) fraction 6; Table S1. Pig (*Sus scrofa*, Uniprot ID UP000008227) specific peptides detected in pork luncheon loaf sample, before and after strong cation exchange (SCX) fractionation (Fraction 1−6). Positive detection (+) indicates the peptide was identified in non-fractionated sample or a corresponding SCX fraction, while (−) indicates absence.

## Abbreviations

The following abbreviations are used in this manuscript:

CAF	Chemically activated fragmentation
CID	Collision-induced dissociation
DDA	Data-dependent acquisition
DIA	Data-independent acquisition
DIA-MS^E^	Data-independent MS/MS acquisition
ELISA	Enzyme-linked immunosorbent assay
FBDA	4-formylbenzene-1,3-disulfonic acid
KMD	Kendrick mass defect
LC-MS/MS	Liquid chromatography-tandem mass spectrometry
MALDI	Matrix-assisted laser desorption/ionization
MALDI-TOF/TOF	Matrix-assisted laser desorption/ionization time-of-flight/time-of-flight
MS	Mass spectrometry
MS/MS	Tandem mass spectrometry
nanoESI	Nano-electrospray ionization
nano-LC-QTOF-MS/MS	Nano-liquid chromatography-quadrupole time-of-flight tandem mass spectrometry
nanoUPLC	Nano ultra-performance liquid chromatography
OD	Optical density
PLGS	ProteinLynx Global Server
PKL	Peak List File
PP-µSPE	Positive-pressure micro solid-phase extraction
qPCR	Quantitative polymerase chain reaction
qTOF	Quadrupole time-of-flight
SCX	Strong cation exchange
SPE	Solid phase extraction
TrEMBL	Translated EMBL database
UniProtKB	UniProt Knowledgebase
