# Supplementary material for: Identification of Species-Specific Peptide Markers in Highly Processed Meat Products Using De Novo Sequencing
Source: Foods. 2026 Jun 26;15(13):2294. doi: 10.3390/foods15132294 (PMC13361223; doi:10.3390/foods15132294)
Supplement: Supplementary file 1 [file foods-15-02294-s001.zip › foods-4386925-supplementary.pdf]

Supplementary Material

Identification of Species-Specific Peptide Markers in Highly Processed Meat Products Using De Novo Sequencing

Renata Biba [1,†], Mihaela Pravica [1,†], Ivana Varenina [2], Nina Bilandžić [2] and Mario Cindrić [1,*]

[1] Laboratory for Bioanalytics, Division of Molecular Medicine, Ruđer Bošković Institute, Bijenička Cesta 54, 10000 Zagreb, Croatia; renata.biba@irb.hr (R.B.); mihaela.pravica@irb.hr (M.P.)
[2] Laboratory for Residue Control, Department of Veterinary Public Health, Croatian Veterinary Institute, Savska Cesta 143, 10000 Zagreb, Croatia; kurtes@veinst.hr (I.V.); bilandzic@veinst.hr (N.B.)
[*] Correspondence: mcindric@irb.hr
[†] These authors contributed equally to this work.

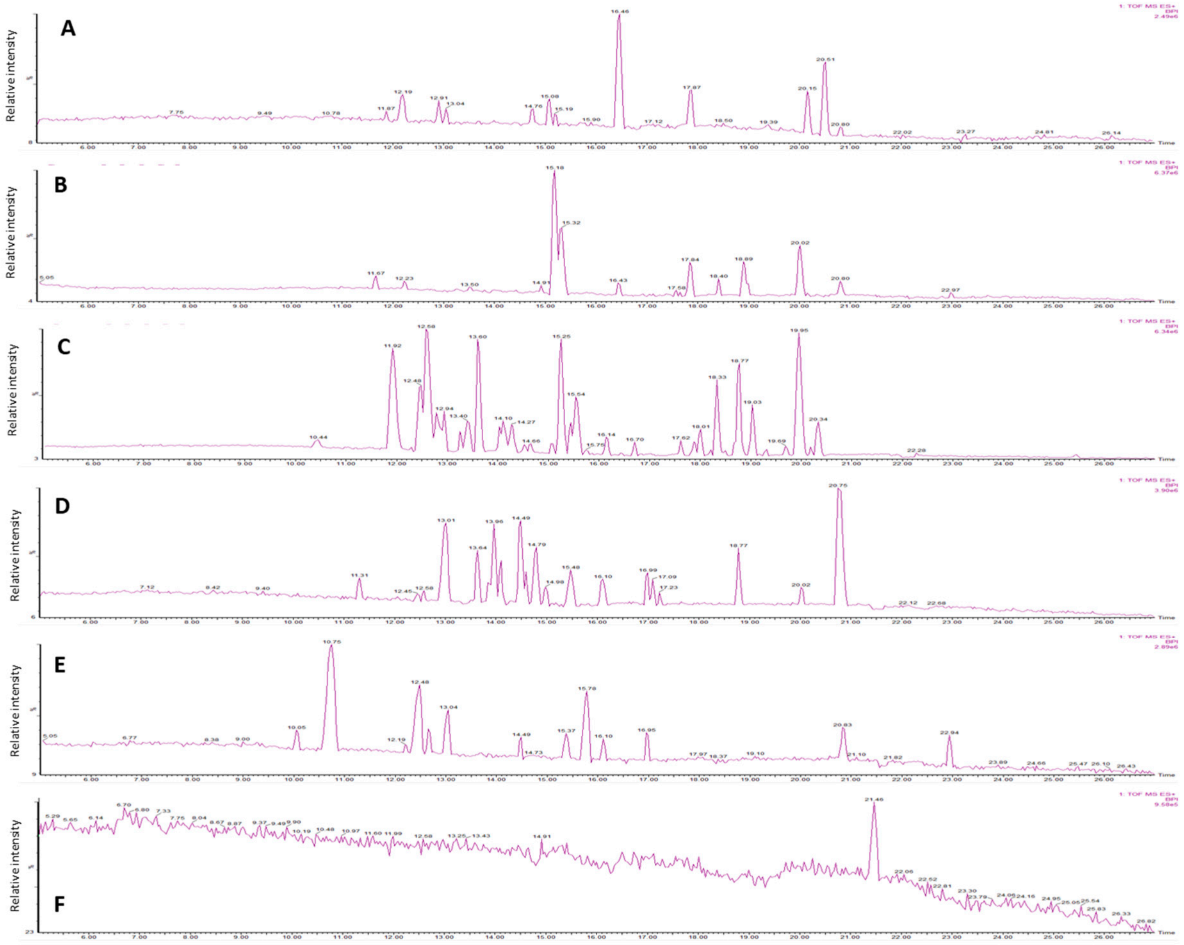

Figure S1. Representative base peak intensity (BPI) chromatograms of FBDA-derivatized peptides obtained from chicken luncheon loaf samples, SCX fractionated, and analyzed by DIA-MS^E in positive ion mode. A) fraction 1, B) fraction 2, C) fraction 3, D) fraction 4, E) fraction 5, F) fraction 6

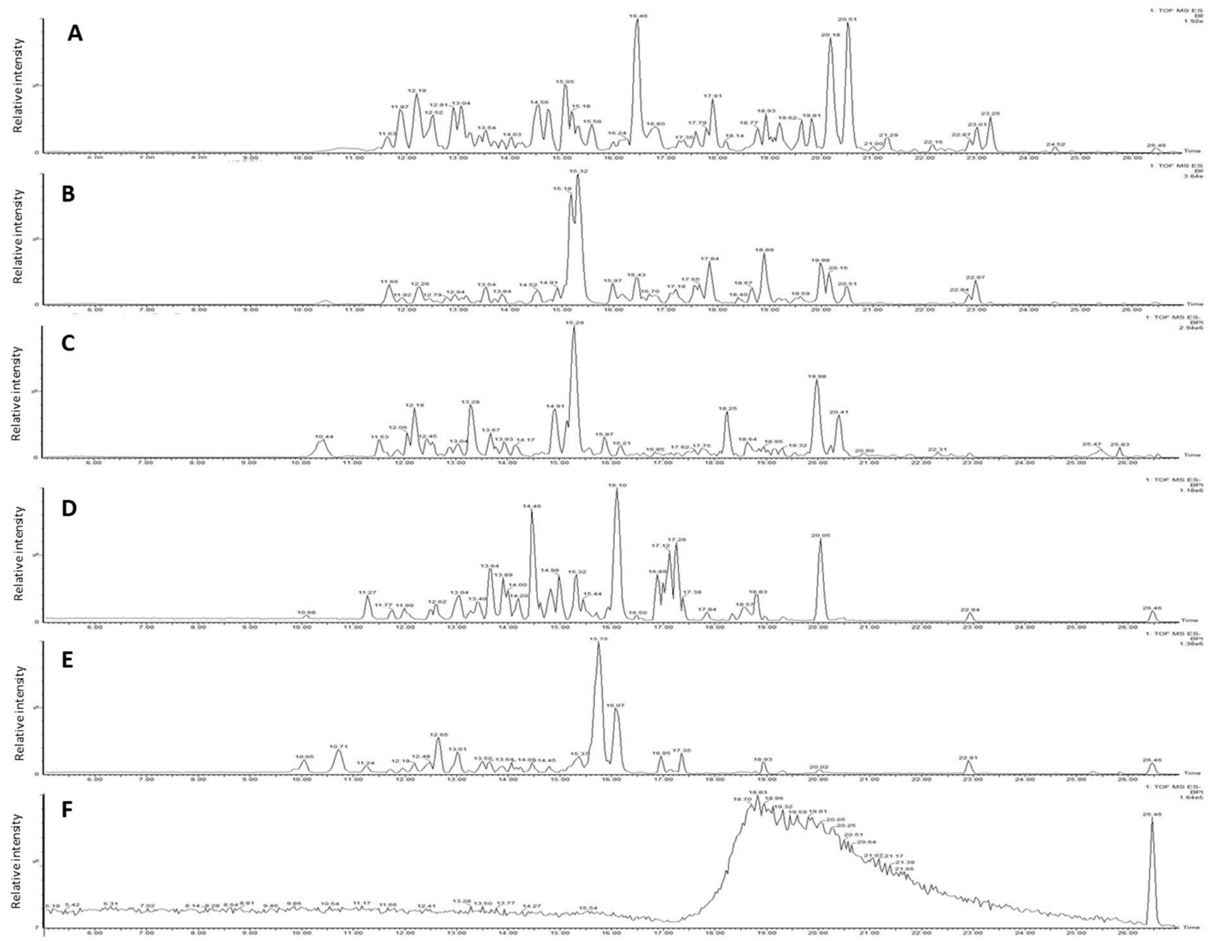

Figure S2. Representative base peak intensity (BPI) chromatograms of FBDA-derivatized peptides obtained from chicken luncheon loaf samples analyzed by DIA-MS^E in negative ion mode. A) fraction 1, B) fraction 2, C) fraction 3, D) fraction 4, E) fraction 5, F) fraction 6

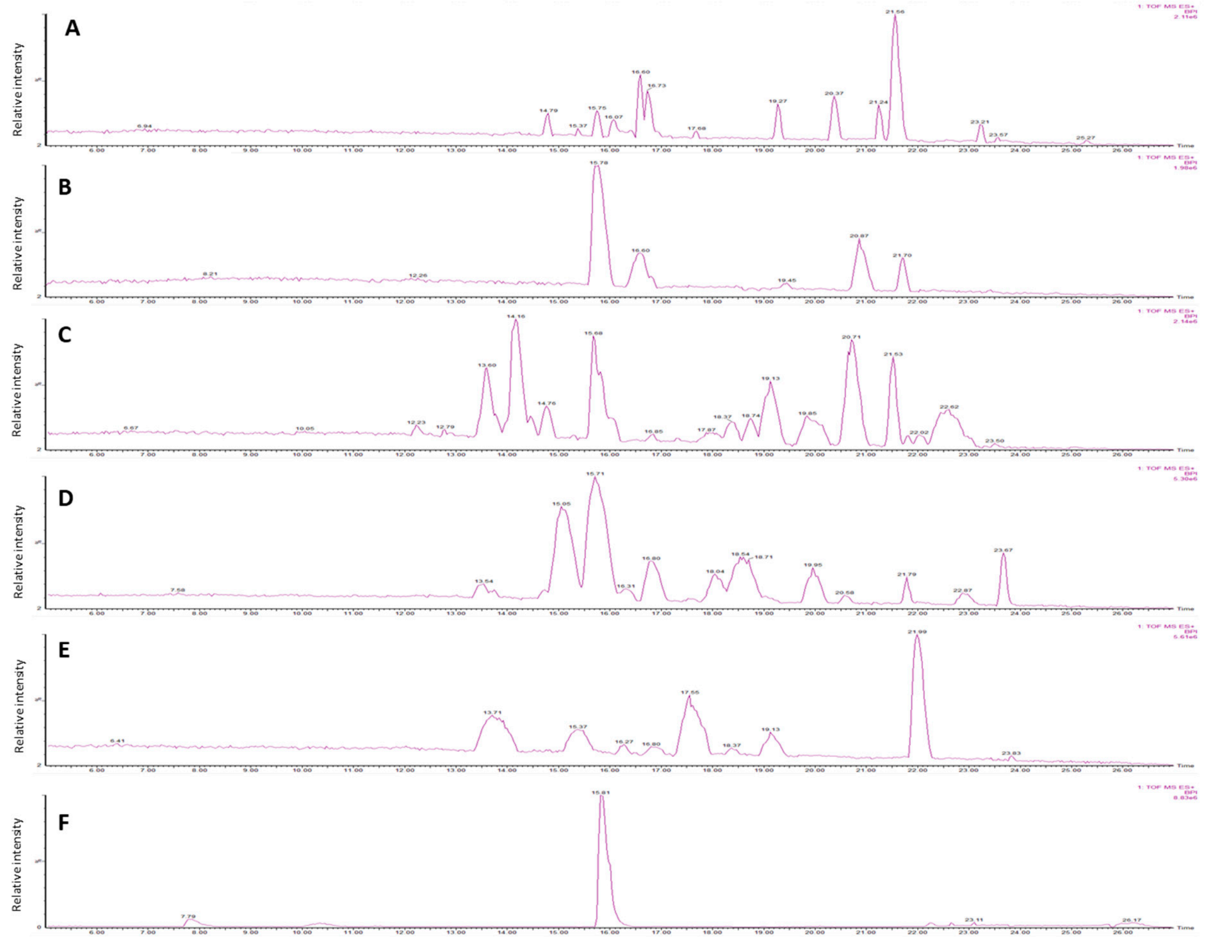

Figure S3. Representative base peak intensity (BPI) chromatograms of FBDA-derivatized peptides obtained from beef luncheon loaf samples, SCX fractionated, and analyzed by DIA-MS^E in positive ion mode. A) fraction 1, B) fraction 2, C) fraction 3, D) fraction 4, E) fraction 5, F) fraction 6

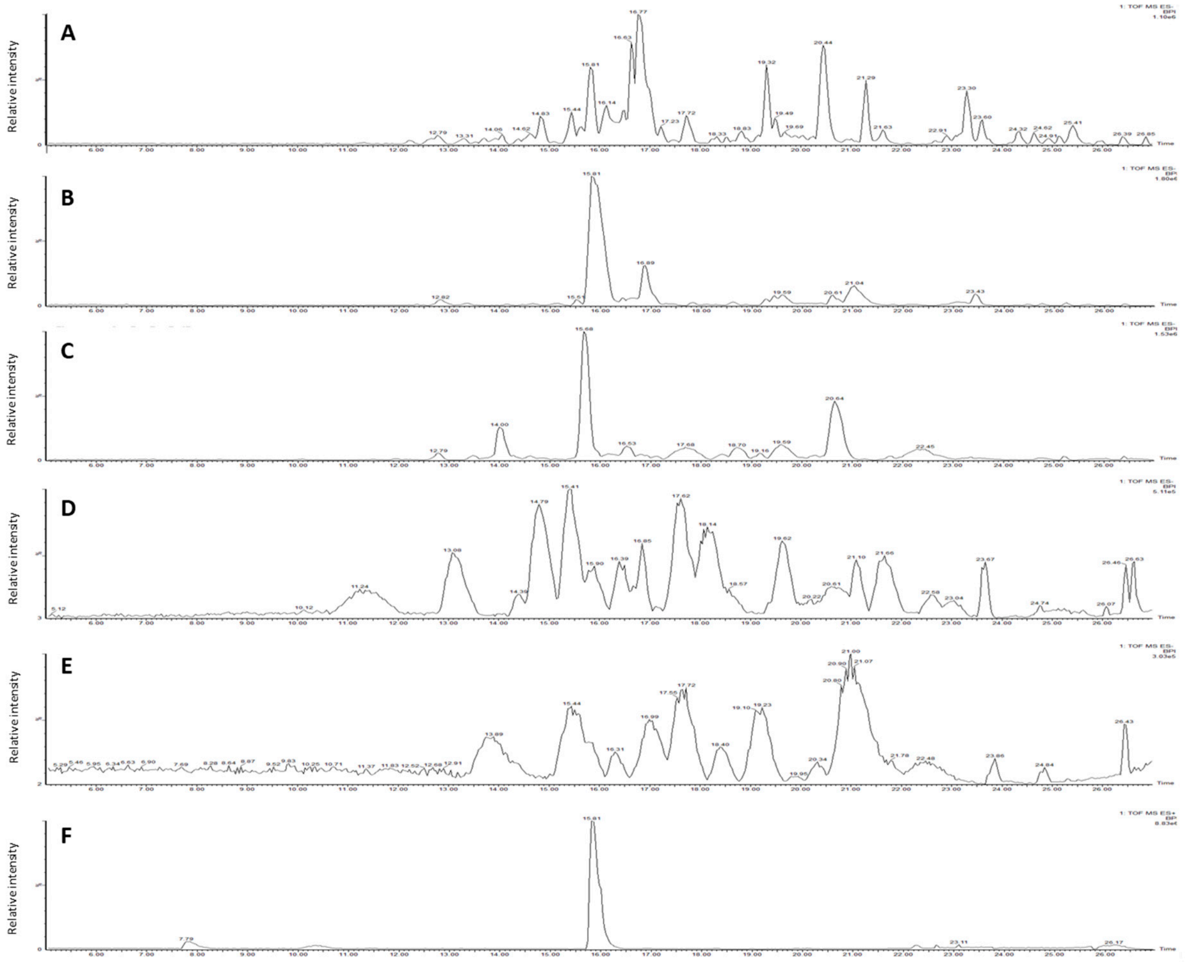

Figure S4. Representative base peak intensity (BPI) chromatograms of FBDA-derivatized peptides obtained from beef luncheon loaf samples analyzed by DIA-MS^{E} in negative ion mode. A) fraction 1, B) fraction 2, C) fraction 3, D) fraction 4, E) fraction 5, F) fraction 6

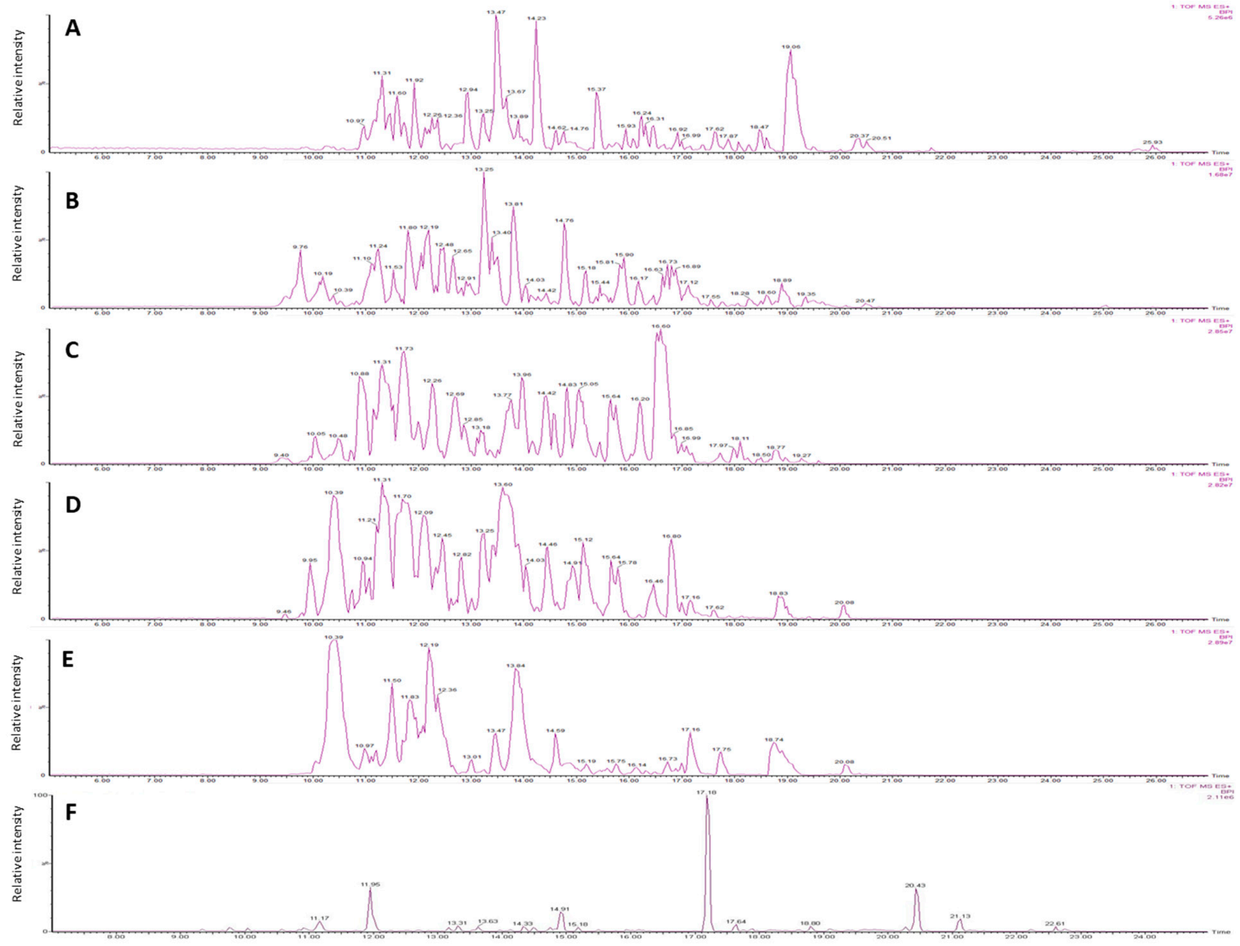

Figure S5. Representative base peak intensity (BPI) chromatograms of FBDA-derivatized peptides obtained from 10% chicken / 90% pork luncheon loaf samples, SCX fractionated, and analyzed by DIA-MS^E in positive ion mode. A) fraction 1, B) fraction 2, C) fraction 3, D) fraction 4, E) fraction 5, F) fraction 6

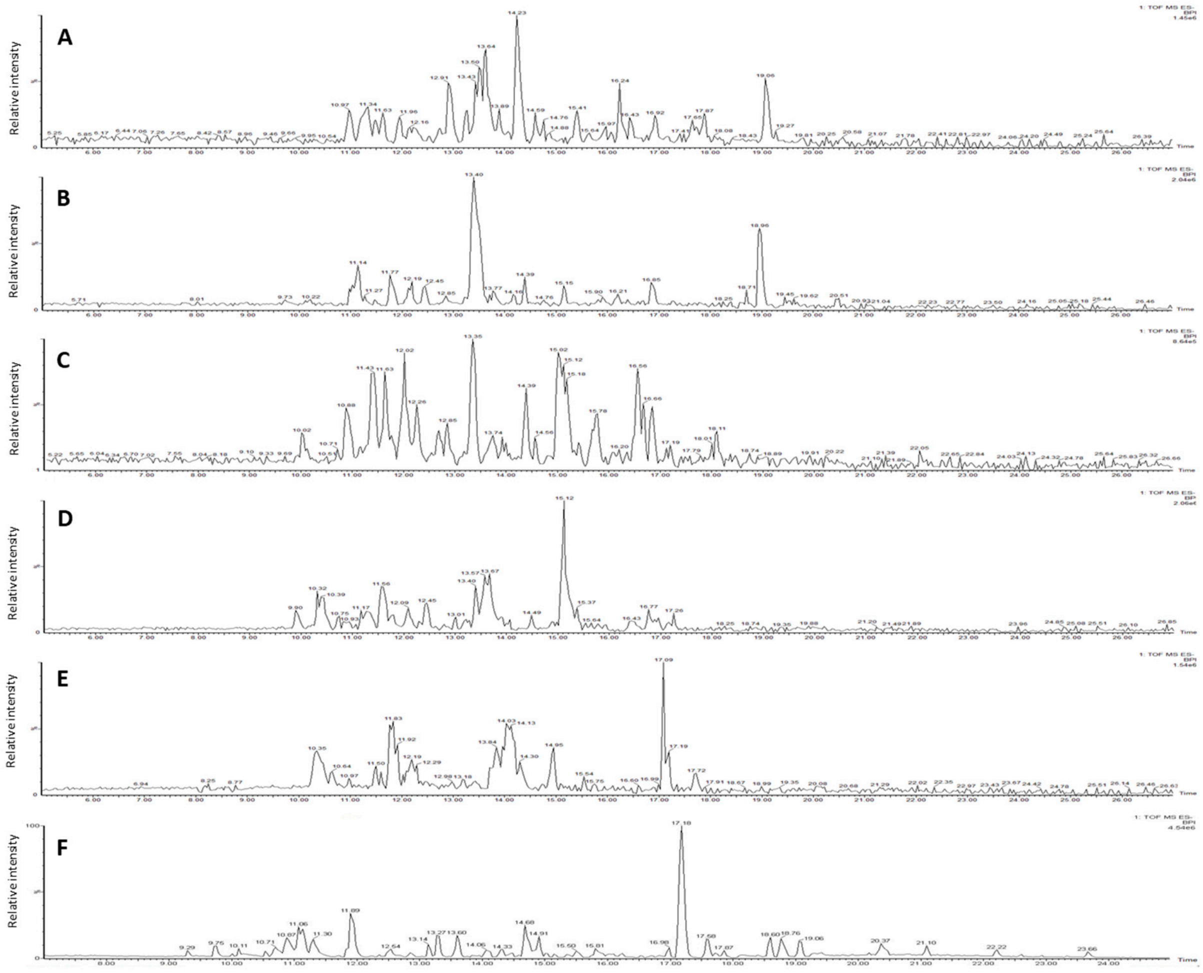

Figure S6. Representative base peak intensity (BPI) chromatograms of FBDA-derivatized peptides obtained from 10% chicken / 90% pork luncheon loaf samples analyzed by DIA-MS^E in negative ion mode. A) fraction 1, B) fraction 2, C) fraction 3, D) fraction 4, E) fraction 5, F) fraction 6

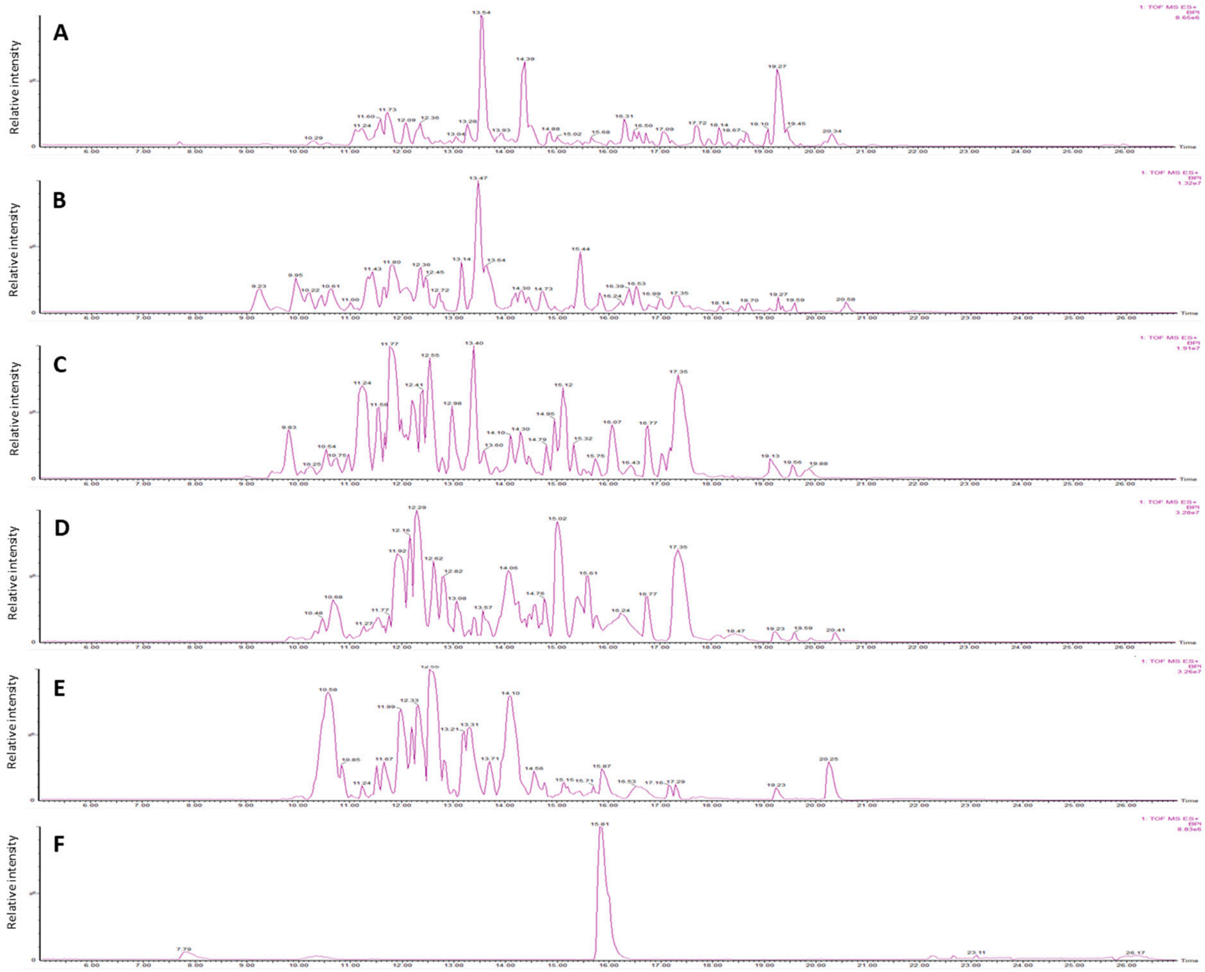

Figure S7. Representative base peak intensity (BPI) chromatograms of FBDA-derivatized peptides obtained from 10% chicken / 90% beef luncheon loaf samples, SCX fractionated, and analyzed by DIA-MS^E in positive ion mode. A) fraction 1, B) fraction 2, C) fraction 3, D) fraction 4, E) fraction 5, F) fraction 6

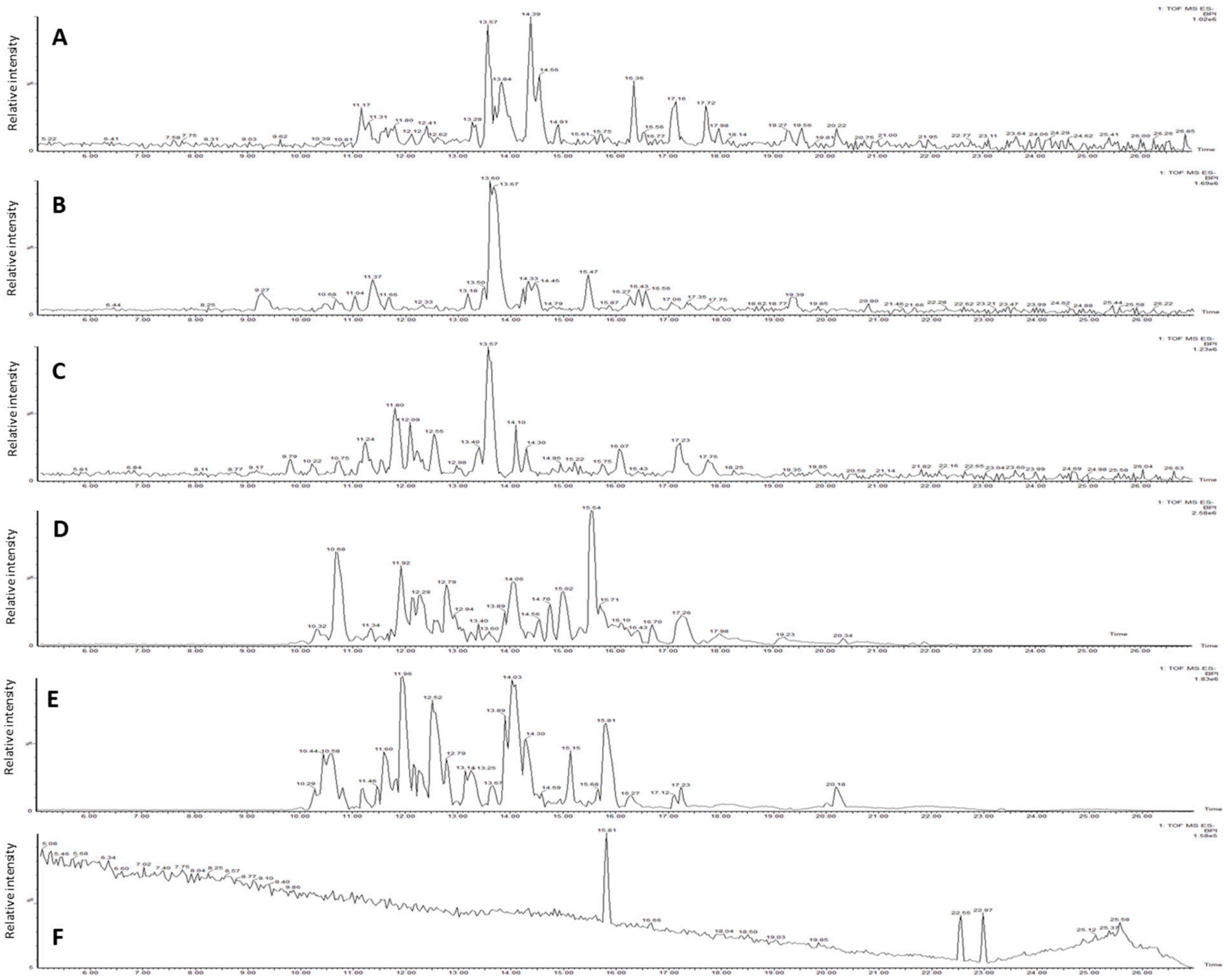

Figure S8. Representative base peak intensity (BPI) chromatograms of FBDA-derivatized peptides obtained from 10% chicken / 90% beef luncheon loaf samples, SCX fractionated, and analyzed by DIA-MS^E in negative ion mode. A) fraction 1, B) fraction 2, C) fraction 3, D) fraction 4, E) fraction 5, F) fraction 6.

Table S1. Pig (*Sus scrofa*, Uniprot ID UP000008227) specific peptides detected in pork luncheon loaf sample, before and after strong cation exchange (SCX) fractionation (Fraction 1−6). Positive detection (+) indicates the peptide was identified in non-fractionated sample or a corresponding SCX fraction, while (−) indicates absence.

Species (UniProt ID)	Protein	Accession No. (UniProtKB/ TrEMBL)	Peptide sequence	Non-fractionated	FR 1	FR 2	FR 3	FR 4	FR 5	FR 6
**Pig - *Sus scrofa* (UP000008227)**	Hemoglobin subunit alpha	A0A4X1UIB6	MFLGFPTTK	+	+	-	-	-	-	-
			VGGQAGAHGAEALER	-	+	-	-	-	-	-
			TYFPHFNLSGSDQVK	+	-	-	-	-	+	+
	Troponin T, fast skeletal muscle	A0A287AFK2	QKYDIINLR	-	+	-	-	-	-	-
	Myosin-2	A0A8D1L3D5	QAYTQQIEELKR	-	+	-	-	-	-	-
	Creatine kinase	A0A8D1L9L8	ALTLEIYKK	+	+	-	-	-	-	-
